# Pathway Analysis Interpretation in the Multi-Omic Era

**DOI:** 10.3390/biotech14030058

**Published:** 2025-07-29

**Authors:** William G. Ryan V., Smita Sahay, John Vergis, Corey Weistuch, Jarek Meller, Robert E. McCullumsmith

**Affiliations:** 1 Department of Neurosciences and Psychiatry, College of Medicine and Life Sciences, University of Toledo, Toledo, OH 43606, USA; 2 Department of Medical Physics, Memorial Sloan Kettering Cancer Center, New York, NY 10065, USA; 3 Department of Environmental and Public Health Sciences, University of Cincinnati, Cincinnati, OH 45221, USA; 4 Department of Computer Science, University of Cincinnati, Cincinnati, OH 45221, USA; 5 Division of Biomedical Informatics, Cincinnati Children’s Hospital Medical Center, Cincinnati, OH 45229, USA; 6 Department of Informatics, Nicolaus Copernicus University, 87-100 Toruń, Poland; 7 Neurosciences Institute, ProMedica, Toledo, OH 43606, USA

**Keywords:** omics interpretation, pathway analysis, gene ontology, embeddings, semantic similarity

## Abstract

In bioinformatics, pathway analyses are used to interpret biological data by mapping measured molecules with known pathways to discover their functional processes and relationships. Pathway analysis has become an essential tool for interpreting large-scale omics data, translating complex gene sets into actionable experimental insights. However, issues inherent to pathway databases and misinterpretations of pathway relevance often result in “pathway fails,” where findings, though statistically significant, lack biological applicability. For example, the Tumor Necrosis Factor (TNF) pathway was originally annotated based on its association with observed tumor necrosis, while it is multifunctional across diverse physiological processes in the body. This review broadly evaluates pathway analysis interpretation, including embedding-based, semantic similarity-based, and network-based approaches to clarify their ideal use-case scenarios. Each method for interpretation is assessed for its strengths, such as high-quality visualizations and ease of use, as well as its limitations, including data redundancy and database compatibility challenges. Despite advancements in the field, the principle of “garbage in, garbage out” (GIGO) shows that input quality and method choice are critical for reliable and biologically meaningful results. Methodological standardization, scalability improvements, and integration with diverse data sources remain areas for further development. By providing critical guidance with contextual examples such as TNF, we aim to help researchers align their objectives with the appropriate method. Advancing pathway analysis interpretation will further enhance the utility of pathway analysis, ultimately propelling progress in systems biology and personalized medicine.

## 1. Introduction

The development of high-throughput multi-omics technologies, such as genomics, transcriptomics, and proteomics, has revolutionized biological research, enabling the exploration of cellular processes with unprecedented depth and scale [1]. These advances have catalyzed the rise of systems biology, an integrative approach that focuses on the interactions within biological systems rather than isolated molecular components [2]. By combining diverse omics layers, researchers are now driving personalized medicine forward, tailoring treatments to individual biological signatures derived from patient-specific data [3]. In this context, pathway analysis has become an indispensable tool for translating omic datasets into actionable clinical insights by targeting key pathways and mechanisms for further investigation and manipulation [4]. As multi-omics data continue to expand, pathway analyses offer a crucial method for gleaning meaningful biological conclusions from raw data, linking molecular changes to functional outcomes across various experimental conditions [5]. This approach advances our understanding of disease pathogenesis and also accelerates the development of targeted therapies [6].

Despite pathway analyses providing instrumental insights into biological functioning, inherent challenges often prevent effective application. Translating these analyses into actionable experimental targets and candidate treatments remains a persistent bottleneck in omics-driven research [7,8]. Further, deciphering clinically relevant mechanisms is difficult given the unpredictable interactions within biological systems [9,10,11,12,13]. Extensive lists of results are often generated. While promising, such results are difficult to curate manually, increasing the risk of bias during pathway selection [14,15,16]. Moreover, even when results may appear promising, validation is frequently hindered due to the variability among experimental models [17,18,19] and the potential mismatch between statistical confidence and biological significance [20,21].

Another key challenge lies in the complexity of new biological data types, such as kinome array data, continually outpacing the capability to be analyzed, leaving researchers without techniques to translate their findings in meaningful ways [22,23]. The diversity of available tools further complicates this process. Varying algorithms and databases may yield inconsistent results, which often makes the harmonization of outputs impractical [24]. Thus, researchers carry the responsibility of evaluating the accuracy and practical utility of selected tools to ensure proper integration of multi-omics data and sensible outputs [25,26]. An example of this is the correct prioritization and validation of selected pathways for targeted therapies [27]. Misinterpretation or incomplete analysis of pathway data may lead to erroneous biological conclusions, undermining the reliability of personalized treatments [28].

Here, we address a critical gap by focusing on the interpretation of pathway analysis results, a novel approach distinct from previous work [29,30,31,32], which primarily examines different methods for performing pathway analyses. We evaluate the strengths, limitations, and practical applicability of various pathway analysis interpretation methods, offering guidelines to optimize their use across diverse research settings. Ultimately, we argue that the future of pathway analysis lies in enhancing automation, scalability, and the development of tools that produce interpretable outputs, bridging the gap between computational predictions and experimental validation.

## 2. Key Challenges to Pathway Analysis

### 2.1. Pathway Annotation

One of the primary issues in pathway annotation arises from how pathways were first identified and named [33]. Pathway names typically reflect the initial experimental conditions in which they were discovered, rather than encompassing their broader roles. A notable example of this is the Tumor Necrosis Factor (TNF) pathway. Despite its name, TNF is not solely a mechanism for tumor necrosis. Early researchers linked TNF with tumor suppression, as necrosis of tumors was observed in vivo under specific pathological experimental conditions [34]. However, subsequent studies revealed TNF as a multipotent cytokine involved in numerous physiological processes, including the innate immune response, inflammation, and apoptosis across many different tissues [35]. This represents an example of a domain-specific anchor bias [36], wherein the initial characterization of a pathway became a fixed reference point influencing subsequent perspectives. With TNF, the original association with tumor necrosis has anchored its perception, overshadowing its other roles in normal physiology. Highlighting this concern, the so-called “TNF pathway” also mediates NMDA receptor activity in neurons and glial cells [37]. Such semantic mismatches obscure a pathway’s true biological functions, perpetuating narrow interpretations and hindering a comprehensive understanding of pathways beyond the conditions in which they were originally discovered [38,39].

Interpreting function is also highly context-dependent, requiring careful consideration of experimental design and biological domain. For instance, in cancer research, activation of apoptosis is typically associated with programmed cell death, the mechanism for eliminating cancerous cells [40]. However, in the brain, similar activation likely indicates synaptic pruning or neurite retraction, which are critical for neurodevelopment and synaptic plasticity [41,42,43]. Similarly, inflammation activated during injury or infection might signal tissue damage and repair processes [44,45], whereas in the brain, these may reflect immune activation in response to neuroinflammation or other neural stimuli [46,47]. A notable example of this context dependence is the NF-κB pathway, which has distinct canonical and non-canonical activation mechanisms. The canonical NF-κB pathway is typically associated with acute inflammatory responses and innate immunity [48], while the non-canonical pathway governs processes like lymphoid organ development and adaptive immune signaling [49]. Misinterpreting activation could lead to flawed conclusions, such as conflating an immune response with developmental signaling or vice versa. Without accounting for the biological context, researchers may draw incorrect conclusions about pathways’ roles, potentially leading to flawed experimental designs or misdirected therapeutic strategies.

Bias, redundancy and overlap in pathway annotation databases also present significant challenges for interpreting enrichment results [47]. Databases like Gene Ontology (GO), Kyoto Encyclopedia of Genes and Genomes (KEGG), Reactome, WikiPathways, and others commonly describe similar biological functions each using slightly different gene sets or interaction details [50]. Within databases, pathways may be annotated as multiple distinct terms with close variations in the genes and regulatory mechanisms involved, complicating interpretation. For example, in GO, “cell adhesion” (GO:0007155) is annotated as the attachment of cells to other cells or the extracellular matrix, while “cell-cell adhesion” (GO:0098609) specifies adhesion between cells only. Similarly, “cell migration” (GO:0016477) refers specifically to directed cell movement, while “cell motility” (GO:0048870) includes directed movement and also broader movements. These subtle distinctions result in overlapping enrichment results, hindering prioritization of the most relevant pathways [51,52].

Structural differences between annotation databases compound these challenges, obscuring shared biological function and contributing to inconsistencies in pathway coverage. For example, overlapping gene sets among those labeled as “Wnt signaling” in KEGG, Reactome, and WikiPathways have significant divergence, with only 73 overlapping genes out of 148, 312, and 135 total genes, respectively, despite annotating the same function [53]. Furthermore, total gene coverage is heavily disparate, with databases like Reactome annotating over 10,000 human genes, while others like WikiPathways cover only about 6000. Study biases further exacerbate this problem, as genes frequently studied in fields like cancer research, where omics is used predominantly, are counterintuitively underrepresented in gene-set annotations [54]. Similarly, GO, originally developed for model organisms, may overemphasize highly conserved cellular processes at the expense of species-specific functions [55]. Despite this stringency, even curated GO annotations have error rates ranging from 18% to 49% [55].

**Figure 1:**
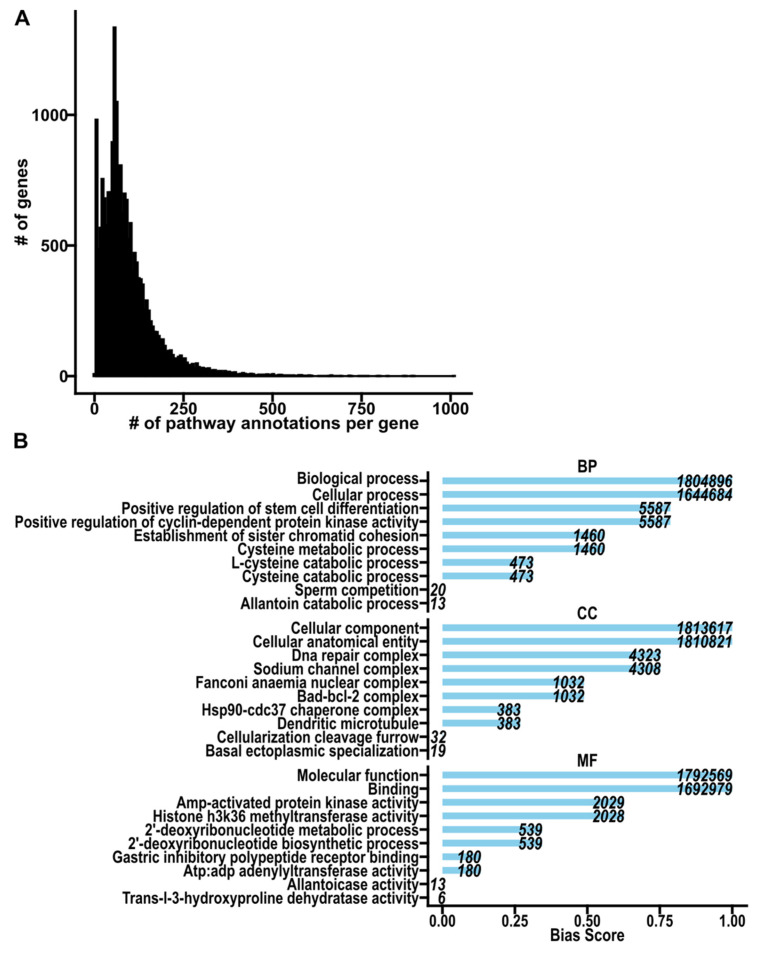
Gene ontology gene-term annotations (GOALL w/IEA, Bader Lab, October 2024). (**A**). Histogram showing the frequency of all annotated genes and the number of pathways, i.e., GO terms, annotated to them. (**B**). Bar plot showing representative terms in each GO subontology after percentile ranking all terms by their cumulative gene annotations (bias score) and using Tukey’s five-number summary. BP: biological process; CC: cellular component; MF: molecular function.

To explore potential biases in annotations, we surveyed gene sets presently annotated to GO terms, i.e., pathways, which illustrated how these challenges may manifest as patterns of redundancy and bias. We found that certain genes, like *transforming growth factor beta 1*, are annotated to over 1000 pathways, while others like *chromosome 6 open reading frame 62* are annotated to just 2 pathways (Table 1, Supplemental Table S1). Moreover, a substantial number of genes, including 611 genes coding for protein products, lack any known annotation entirely (Table 2). Therefore, if a researcher were to investigate the function of any of these unannotated genes (Supplemental Table S2) using GO, they would be excluded from their analyses. These disparities result in highly skewed gene coverage, where a small subset of genes dominate annotations (Figure 1A). Similarly, a handful of highly annotated pathways disproportionately account for most of all known gene–pathway associations (Figure 1B). These patterns highlight the broader challenges of redundancy and bias in practice, which obscures biological significance of underrepresented genes and hinders the prioritization of relevant results by researchers. Efforts to mitigate these issues, such as set theory-based approaches to reduce overlap, have shown promise but require further refinement to ensure comprehensive and equitable representation within annotation databases [56]. Awareness of these domain-specific challenges is essential to improve the clarity and utility of pathway annotations.

**Table 2:** **Locus types of all HGNC genes with zero gene ontology annotations (GOALL w/IEA, Bader Lab, October 2024).** HGNC: HUGO Gene Nomenclature Committee.

Locus Type	Count
pseudogene	13,940
RNA, long non-coding	5640
RNA, micro	1912
gene with protein product	611
RNA, transfer	591
RNA, small nucleolar	568
immunoglobulin pseudogene	202
readthrough	143
RNA, cluster	119
fragile site	116
endogenous retrovirus	92
T cell receptor gene	67
RNA, ribosomal	58
immunoglobulin gene	55
RNA, small nuclear	51
region	46
unknown	46
T cell receptor pseudogene	38
RNA, misc	29
virus integration site	8
complex locus constituent	6
RNA, vault	4
RNA, Y	4

**Table 3:** **Overview of pathway analysis interpretation tools and their features.** This table lists various tools categorized by their method of analysis (e.g., semantic, network, embedding), detailing their year of release, description, access platform, supported databases, and visualization capabilities. This comparison serves as a comprehensive resource for researchers to identify tools best suited for their specific pathway analysis needs.

Tool	Year	Method	Access	Database	Visualization	Description
REVIGO [57]	2011	**Semantic**	Web	GO	Scatterplots, Interactive graph, tree maps	Summarizes GO term list using Semantic Similarity and clustering
clusterProfiler [58]	2013	**Semantic**	R package	GO, KEGG, DO	Dot plot	Enrichment analysisfor GO/KEGG terms and visualization
ReCiPa [52]	2018	**Semantic**	R package	KEGG, Reactome	Data tables	Controls redundancy in pathway databases
GOGO [59]	2018	**Semantic**	Web, Perl	GO	Data tables	Calculates semantic similarity of GO terms using improved algorithms
FunSet [60]	2019	**Semantic**	Web, Standalone	GO	2D plots	Performs GO enrichment analysis with interactive visualizations
GeneSetCluster [61]	2020	**Semantic**	R package	Any	Network graph, dendrogram, heatmap	Groups gene sets post analysis based on shared genes
GOMCL [62]	2020	**Semantic**	Python	GO	Heatmap, Network graph	Clusters GO terms using Markov clustering algorithm
GoSemSim [63]	2020	**Semantic**	R package	GO	Data tables	Computes semantic similarity among GO terms for comparison
GO-FIGURE! [15]	2021	**Semantic**	Python	GO	Scatterplot	Visualizes GO term similarity with custom scatterplots
Simplify Enrichment [64]	2022	**Semantic**	R package	GO	Heatmap	Clusters with a unique binary cut algorithm.
iDEP [65]	2018	**Semantic**	Web, R package	GO, KEGG, Reactome	Tree, Heatmap, Network Graph	Web app for transcriptomics And pathway exploration
DAVID [66]	2009	**Semantic**	Web, REST API	KEGG, Any	Tabular, Barchart, Network graph	Enrichment analysis with functional annotation clustering
g:Profiler [67]	2007	**Semantic**	Web, R package	KEGG, Reactome, WikiPathways, Any	Dot plot, Tabular, Network graph	Orthology-aware enrichment analysis and clustering
RICHNET [68]	2019	**Network**	R protocol	MSigDB	Network graph	Automated gene-set network creation
EnrichmentMap [69]	2019	**Network**	Cytoscape	Any	Interactive network	Detailed enrichment mapping
Gscluster [70]	2019	**Network**	Web, R Package	MSigDB	Interactive network	Network-weighted gene-set clustering integrating PPI data
aPEAR [71]	2019	**Network**	R package	Any	Network graph	Clustering with automated naming
GeneFEAST [72]	2023	**Network**	Web, Python	Any	Heatmap, Dot plot, Upset plot	Highlights multi-enrichment genes
vissE [73,74]	2023	**Network**	R package	MSigDB, Any	Network graph	Visualizes higher-order interactions
pathlinkR [75]	2024	**Network**	R package	Reactome, MSigDB, InnateDB	Network graph, Volcano plot, Dot plot	Integrated PPI network construction
Pathview [76]	2017	**Network**	Web, R Package, REST API	KEGG	Network Graph	Visualizes and maps data onto KEGG pathways
PAVER [77]	2024	**Embedding**	Web, R package	Any	UMAP, Heatmap, Dot plot	Embedding-based clustering with UMAP for clear pathway visualization
Mondrian- Map [78]	2024	**Embedding**	Python	WikiPathways	Mondrian Map	Embedding visualizations highlighting pathway interactions and crosstalk
GOsummaries [79]	2015	**Word Cloud**	R package	GO	PCA, Boxplot	Visualizes GO analyses as word clouds and overlays results
genesetSV [80]	2023	**Game Theory**	Python	KEGG, MSigDB	Scatterplot	Uses Shapley values for ranking and reducing pathway sets
Archetype- Discovery [81]	2024	**Non-negative** **Matrix** **Factorization**	MATLAB	MSigDB, Any	Radar, Scatter- and Boxplot, Heatmap	Derives compact archetypal gene-set patterns and their pathway associations

### 2.2. Visualizing Pathway Findings

Another critical challenge in pathway analysis is effectively visualizing high-dimensional data, which is essential for interpreting results and communicating findings [7,10]. The complexity of multi-omics datasets often makes illustrating informative relationships between data difficult [25]. Without specialized computational expertise, this complexity may hinder an experimental biologist’s ability to derive meaningful conclusions [82]. Visualization tools must therefore balance simplifying data for clarity while retaining necessary detail for accurate interpretation [83]. Moreover, visualization is further complicated by the aforementioned redundancy, leading to visual clutter that obscures key findings. Heatmaps, Uniform Manifold Approximation and Projection (UMAP) plots, and network-based plots address these challenges by offering more intuitive representations. Notably, these approaches all have limitations, such as artifacts introduced by dimensionality reduction or network overcrowding and sparsity [69,84]. A lack of standardized and widely accepted visualization practices exacerbates this challenge, leaving researchers with fragmented outputs. Thus, developing more effective and user-friendly visualization strategies remains a key priority for advancing pathway analysis and improving accessibility to a broader research audience.

### 2.3. Limitations to Pathway Analysis Utility

Despite the potential of pathway analysis to generate new insights, it often falls short in providing *actionable* leads, resulting in cases where results are uninformative. In studies using gene expression data, analyses may overrepresent genes in canonical pathways, such as “immune function,” even when biologically irrelevant to the experimental conditions [85]. For example, in an RNAseq study of postmortem DLPFC tissue exploring gene co-expression networks related to schizophrenia [86], eye development was identified within astrocyte modules. Such results lack readily apparent insights into schizophrenia-specific biological mechanisms. Similarly, pathways associated with learning and memory, highlighted in neuron modules, presented challenges as their relevance to schizophrenia risk and clinical state is extremely vague. Such “pathway fails” are not uncommon. For example, the kynurenine pathway’s role in psychiatric conditions such as schizophrenia and major depressive disorder is well characterized [87]. However, the precise mechanisms by which it contributes to changes in cognitive function remain poorly understood. This lack of clarity complicates the development of targeted therapeutic interventions, exemplifying how even well-studied pathways can yield results that are uninformative for real-world translational applications. These instances highlight a common pitfall in pathway analysis since statistical significance does not necessarily equate to biological relevance [20,21].

However, not all analyses fall short; there are notable examples of “pathway successes” that have led to significant clinical advancements: nearly two-thirds of recent FDA-approved drugs were shown a priori that their gene or protein targets had a significant phenotypic association with the targeted disease [88]. Anifrolumab, an IFNAR1 antagonist approved for systemic lupus erythematosus (SLE), lacks direct association with SLE. However, variants in TYK2—a kinase that physically interacts with IFNAR1—have a pathogenic role acting via the TYK2/JAK pathways in a pathway association study [89,90]. Similarly, PCSK9, a target for hyperlipidemia therapies, was identified through pathway analysis despite lacking direct genetic association with the disease; instead, it was implicated via protein interaction networks whose interacting nodes were enriched for hyperlipidemia-related pathways [91]. These successes underscore the potential of pathway analysis to yield actionable insights, even as challenges persist in ensuring biological relevance and clinical utility. Selecting appropriate analysis methods and interpreting results within the appropriate biological context is crucial to avoid uninformative or misleading conclusions.

### 2.4. Discrepancies in Molecular Biology Mislead Validation

Traditionally, mRNA (i.e., gene) expression levels and protein abundance have been relied on as proxies for biological activity [92,93,94]. While mRNA levels are often used as convenient inferences of protein expression, substantial evidence shows that mRNA and protein abundances are not well-correlated [95,96]. Gene expression levels often do not correlate with protein abundance, and protein abundance does not necessarily reflect functional activity due to factors such as post-translational modifications, protein–protein interactions, and subcellular localization [97,98]. These discrepancies present significant challenges in pathway analyses based solely on transcriptomics or proteomics data, as they may not fully reflect functional states of biological pathways. Researchers unaware of these limitations risk drawing incomplete conclusions about cell processing and signaling [99]. To address this challenge, there has been a shift toward functionally informed methods [100,101,102,103], such as phosphoproteomics and kinome reporter phosphopeptide arrays. These methods provide a functional view of the genes and pathways being studied, moving away from simply observing there is too much or too little of a gene product [104]. By capturing the dynamic nature of biological systems, these approaches may mitigate the inherent pitfalls of traditional omics-driven pathway predictions and enhance the overall reliability of pathway analyses [105].

The cell subtype context also presents unique challenges, as scRNAseq datasets are dominated by dropout events that create sparse expression matrices and obscure true signals [106]. Limited mRNA detection from rare cell populations also introduces selection bias into downstream analyses [107]. Enrichment tools originally developed for bulk RNAseq data therefore risk misclassifying dropouts as biological absence, or inflating significance [108]. Although imputation strategies can fill dropouts, they may amplify technical variation and wash out genuine biological heterogeneity [109]. Consequently, dropout-aware methods are more reliable than traditional approaches [110,111]. Despite these advancements, purpose-built methods require careful consideration of input data quality, outliers, and pathway complexity [112,113]. Collectively, these challenges highlight the need for next-generation, single-cell-specific pathway tools that explicitly model technical noise, accommodate rare cells, and exploit multi-omic integration to minimize “pathway fails” [114].

### 2.5. Research Data Mismanagement

While effective management of datasets is a cornerstone of rigorous and reproducible science, it remains fraught with challenges. The prevalence of data-management errors, such as coding mistakes and ambiguous documentation, undermines scientific reliability and leads to numerous article retractions [115]. These errors, which may occur at any stage of the research workflow, waste resources and highlight the vulnerability of current systems to human fallibility [116]. Retractions due to honest mistakes have also caused immense personal stress and professional consequences for researchers, prompting calls to improve data workflows and prevent similar errors in the future [116]. Emerging frameworks like FAIR (Findable, Accessible, Interoperable, Reusable) data principles aim to address these issues by promoting robust, scalable, and automatable systems that ensure interoperability, standardization, and accessibility of data [117]. By integrating multi-omics datasets into unified frameworks, these principles can enhance the efficiency and reliability of pathway analysis at scale [7,118,119]. As these tools evolve, their capacity to offer high-confidence outputs that align with real-world applicability becomes increasingly important.

## 3. Methods for Pathway Analysis Interpretation

### 3.1. Semantic Similarity Based Methods

Semantic similarity-based methods are a foundational approach in pathway analysis interpretation, designed to quantify the functional relationships among complex annotation terms. For example, semantic similarity-based methods are often applied to interpret the extensive and often redundant lists of GO terms [120]. These methods leverage the hierarchical structure of GO, where terms are organized into parent–child relationships that span from general biological processes to gradually more specific functions [121]. By calculating a “semantic similarity” score between terms, these methods identify and group related GO terms [57], reducing redundancy and enabling researchers to focus on overarching biological themes.

The calculation of semantic similarity typically involves information content metrics based on shared ancestors within the GO hierarchy, reflecting the functional overlap between terms [122]. For instance, terms that share a highly specific ancestor within the GO tree yield higher similarity scores due to their closer functional relationship. Popular metrics include Resnik’s and Lin’s similarity measures, which quantify similarity by evaluating the specificity of shared ancestors and their positions within the GO structure [59]. Tools like clusterProfiler implement these methods by grouping semantically similar GO terms into clusters. Representative terms are selected based on similarity scores and user-defined thresholds, with results visualized through scatter plots or dot plots for clarity [58]. This grouping provides a summarized and non-redundant list of terms, making interpretation of large results more feasible.

However, these methods are inherently tied to the GO framework and can fail to generalize to other databases like KEGG, Reactome, or WikiPathways [121,123]. Additionally, some studies suggest that these similarity scores may not fully capture the nuanced meanings of GO terms, highlighting an area for further refinement [124]. While the combination of functional clustering and visualization described here offers a powerful means of simplifying GO term analysis, new developments are needed to broaden applicability and enhance interpretive precision.

### 3.2. Network-Based Methods

Network-based methods provide a powerful approach for pathway analysis by visualizing interconnected networks, where pathways are represented as nodes, and edges denote their shared genes or similar functional annotations [75]. These methods rely on graph theory principles to quantify relationships between pathways based on shared content, using metrics like gene overlap, Jaccard index, or semantic similarity [68]. This network structure captures relationships between pathways, identifies related terms, and reveals broad biological themes that may be obscured in list-based approaches.

Clustering algorithms, such as modularity-based or hierarchical clustering, group these pathways into distinct modules based on their connectivity, reducing redundancy and highlighting cohesive functional groups [70]. In pathway networks, nodes with above-average connections, or “hubs,” represent key biological functions that interact in communities of multiple pathways, potentially indicating regulatory roles [72]. Visualization techniques position these pathways close together, with edge weights reflecting the strength of their relationships, emphasizing direct and indirect associations across broader biological processes [71].

Tools like EnrichmentMap enhance these analyses by constructing pathway networks based on gene overlap, using significance thresholds to reduce visual complexity and emphasize biologically relevant connections [69]. These clustering and visualization approaches allow researchers to visualize enrichment results using maps to highlight major biological processes and their relationships. Similarly, Visualization of Set Enrichment (vissE) extends the utility of network-based methods by integrating data across modalities, including single-cell and spatial transcriptomics [73,74]. vissE clusters pathways based on their content similarity and links them to specific cellular phenotypes, providing insights into their interactions within complex biological contexts.

Together, these approaches offer researchers a dynamic, systems-level framework for interpreting pathway data by revealing functional relationships at the network level, clustering pathways into interpretable communities, and accordingly simplifying large datasets for actionable insights.

### 3.3. Embedding Based Methods

Embedding-based methods offer a cutting-edge approach to pathway analysis by transforming biological entities, like pathways, into high-dimensional vectors, known as embeddings [125,126]. These embeddings numerically encode the semantic meaning of pathways, capturing complex relationships among genes and pathways within the context of large biomedical datasets. Originating in natural language processing, embeddings are widely used to represent words in hundreds of numerical dimensions, allowing models to mathematically quantify relationships between concepts (e.g., “Proteome − Protein + Kinases = Kinome”) [127]. In biomedical research, this technique has become instrumental for clustering, visualization, and predictive modeling [128].

In pathway analysis interpretation, embedding-based methods reduce redundancy and enhance interpretability by grouping pathways based on their semantic similarity [124]. Tools like Pathway Analysis Visualization with Embedding Representations (PAVER) leverage cosine similarity between embeddings to cluster similar pathways and identify a “most representative term” (MRT) for each group based on the average embedding [77,129,130]. This results in streamlined and summarized output, which is especially useful for managing large datasets. PAVER also employs dimensionality reduction techniques, such as UMAP, to convert high-dimensional embeddings into two-dimensional visualizations. These layouts visually group related pathways while preserving semantic relationships, enabling researchers to more easily identify biological themes and generate publication-ready visualizations [131,132].

Another innovative tool, MondrianMap, draws inspiration from abstract art to spatially arrange pathways on a grid, where proximity reflects functional similarity, and color indicates regulatory states, such as upregulation or downregulation [78]. This intuitive visualization method facilitates the exploration of functional clusters and crosstalk patterns across complex datasets. By going beyond traditional node–edge diagrams or heatmaps, MondrianMap provides an interactive and visually accessible representation of results.

While embedding-based methods are promising, they are limited by the information encoded in annotation descriptions or hierarchical structures [131]. These pre-trained language models can capture broad semantic relationships, but they may lack context-specific details [125]. As these models evolve, embedding-based approaches are likely to become even more sophisticated and scalable for interpreting pathway analysis results across diverse datasets.

### 3.4. Applications of Tools for Pathway Interpretation

The practical utility of exemplar pathway analysis tools can be best appreciated through real-world case studies that illustrate the impact of their specific feature set on advancing biological research.

**clusterProfiler**: clusterProfiler is widely used as a standard for pathway enrichment analysis and visualization in many fields. Chen et al. employed clusterProfiler to identify differentially expressed genes in acute myocardial infarction, identifying key enrichment of cytokine–cytokine receptor interaction and TNF signaling via GO and KEGG pathway analyses [133]. Jia et al. applied clusterProfiler to distinguish unique gene expression profiles between luminal A and basal-like subtypes of breast cancer, identifying pathways involved in subtype-specific progression and novel therapeutic targets, such as neuromedin U receptor 1, neural cell adhesion molecule 1, and STIL centriolar assembly protein [134]. In neurodegenerative research, Niu et al. utilized clusterProfiler to study differentially expressed genes in varying stages of Alzheimer’s disease, uncovering the role of mitochondrial components and proteasome subunits in disease progression [135]. Additionally, Gamazon et al. employed clusterProfiler in a multi-tissue transcriptome study to link gene expression with neuropsychiatric traits, demonstrating its utility in mapping complex genetic influences across brain and non-brain tissues [136]. clusterProfiler’s semantic similarity-based method was particularly valued in these studies for its simplicity, ease of use, and capability to effectively identify key pathways in diverse datasets with minimal computational overhead. However, despite its strengths, the semantic similarity-based method of clusterProfiler may seemingly fail to be effective with the inherent bias and redundancy in annotation databases used like GO or KEGG, potentially leading to the overrepresentation of certain pathways while underrepresenting others with biological relevance. Additionally, the tool’s reliance on predefined annotation databases limits its applicability in contexts where novel, poorly annotated, or species-specific processes are of interest, potentially overlooking critical insights in less-studied biological systems.

**vissE**: vissE excels in handling complex multi-omics data, enabling researchers to identify pathway relationships within diverse contexts at the network level. Kulasinghe et al. utilized vissE to analyze transcriptomic profiles of cardiac tissues from patients who succumbed to SARS-CoV-2. Visualization of enriched pathways related to DNA damage and immune responses was able to pinpoint the molecular impact of COVID-19 on cardiac health [137]. In immune research, Dalit et al. applied vissE to map divergent cytokine and transcriptional signatures across T follicular helper cell populations, revealing how different signaling environments guide immune cell heterogeneity and B cell output during various pathogen exposures [138]. In colorectal cancer, Lee et al. leveraged vissE to explore serotonin-mediated signaling, identifying key interactions linked to tumor growth suppression through ERK signaling [139]. In these studies, vissE’s network-based approach facilitated the identification of pathway–pathway relationships and communities, allowing for a deeper understanding of the complex interactions they observed. However, despite its ability to explain these higher-order phenotypic patterns, vissE’s reliance on network-based visualization can become unwieldy when dealing with highly interconnected or very large datasets, potentially leading to information overload and obscured insights for researchers lacking advanced computational expertise. Moreover, the tool’s dependence on comprehensive input data from multi-omics experiments may amplify the impact of incomplete datasets or biases within them, thereby influencing the reliability and interpretability of the visualized pathways in these specific research contexts.

**PAVER**: PAVER’s embedding-based method, coupled with UMAP visualizations, has proven effective in simplifying complex datasets and revealing critical biological insights. In the brain, Nguyen et al. used PAVER to analyze transcriptomic and kinomic data from mice exposed to pyrethroid pesticides during development, uncovering disruptions in pathways related to MAP kinase signaling and circadian rhythms that may underlie neurodevelopmental disorders [140]. Similarly, Curtis et al. used PAVER to integrate metabolomic and transcriptomic data from the brains of male mice developmentally exposed to deltamethrin, effectively visualizing pathway clusters related to folate biosynthesis, dopamine synapses, and MAPK signaling to highlight the multi-modal impact of environmental exposure on adult brain metabolism [141]. O’Donovan et al. characterized transcriptional changes in the orbitofrontal cortex across psychiatric disorders such as schizophrenia and bipolar disorder, identifying immune-related functions and sex-specific gene expression patterns that distinguished diagnoses and contributed to understanding disease mechanisms [142]. In toxicology research, Hu et al. applied PAVER to interpret kidney transcriptomics in mice exposed to microcystin-LR, demonstrating its use in identifying pathways modulated by probiotic treatment, which offered protective effects against toxin-induced damage [143]. In these studies, PAVER simplified interpretation by visually identifying similar clusters and highlighting functional groups in their multi-omic datasets. However, PAVER necessitates the use of pre-computed embeddings, which may limit flexibility in real-time analyses or exploration of novel datasets. Additionally, the tool’s effectiveness is heavily dependent on the quality and diversity of the input datasets, which may restrict utility in studies where such data are incomplete or unbalanced, potentially impacting the robustness of its pathway clustering and functional interpretations.

Collectively, these tools highlight how different interpretation methods cater to distinct research needs. By choosing the right tool for generating interpretations and deliverables of enrichment results, researchers may maximize the impact of their pathway analyses on the understanding of complex biological systems they study.

### 3.5. Choosing the Right Tool for Your Research

Before selecting any pathway interpretation strategy, researchers must first confirm that the raw data have been rigorously pre-processed, e.g., library-size normalization [144], dropout-aware imputation for zero-inflated single-cell data [145], and cross-study batch-effect correction [146], in order to ensure reproducibility and prevent technical noise from propagating into “pathway fails.” Choosing the most appropriate pathway analysis method then depends on aligning one’s research goals with the specific strengths and limitations of each approach available. The semantic similarity-based, network-based, and embedding-based methods qualitatively compared in Table 2 below may guide researchers in selecting the method that best fits their study’s objectives.

Visualization quality and usability differ across methods. Semantic similarity-based methods, as seen with clusterProfiler, provide straightforward and accessible output of data, such as via bar charts, for researchers seeking simple visualization. Network-based methods, such as vissE, offer interactive and detailed visualizations that map complex relationships between pathways, ideal for the exploration of interrelations at a deeper level. Embedding-based approaches, such as those employed by PAVER, excel in generating high-quality visualizations to simplify clusters for clearer interpretation.

Ease of use varies as well. Semantic similarity-based methods offer simple workflows, appealing to experimental biologists who are new to bioinformatics. In contrast, network-based methods can demand more technical expertise, appealing to researchers who are comfortable navigating intricate visualizations and data relationships. Embedding-based methods generally automate visualization, requiring minimal input, which is beneficial for quick insights and user-friendly experiences.

Effectiveness in handling redundancy and integrating multi-omics data also sets these methods apart. Semantic similarity-based methods, while excellent at reducing GO term redundancy, are more limited when integrating non-GO data. Network-based approaches stand out in integrating multi-omics data by mapping interconnected pathways and revealing functional interactions across different biological layers. Embedding-based methods reduce redundancy effectively by clustering similar pathways, facilitating interpretation of high-dimensional datasets.

Computational efficiency and scalability are further practical considerations. Semantic similarity-based methods are lightweight and run efficiently for smaller-scale analyses. Network-based methods, however, can be more computationally demanding, especially when visualizing extensive networks or handling dense data. Embedding-based methods are often efficient and scalable, making them suitable for large-scale studies involving dimensionality reduction.

Accessibility also varies across methods. Semantic similarity-based methods are widely accessible as R packages or command-line tools with strong community support. Network-based methods might require specific software installations but are supported by active, albeit more niche, user communities. Embedding-based tools are often web-based with minimal setup requirements.

In summary, semantic similarity-based methods are most appropriate for GO-focused studies requiring straightforward analysis. Network-based methods are best suited for complex analyses that need detailed mapping of interactions. Embedding-based methods are ideal for those seeking quick, visually intuitive summaries and data-driven redundancy reduction. By understanding the unique features of each method type (Table 4), researchers can better align their interpretation strategies with their study goals. This thoughtful approach provides a guide for future advancements and adaptations in pathway analysis interpretation as the field continues to evolve.

## 4. Conclusions and Future Directions

This review highlights the strengths and limitations of the three main pathway analysis interpretation methods: semantic similarity-, network-, and embedding-based approaches. Each method offers unique advantages, from the straightforward, GO-focused analyses provided by semantic similarity-based techniques, to the detailed interaction maps facilitated by network-based methods, as well as the high-quality, visually intuitive outputs of embedding-based methods. However, no single approach is without limitations. Challenges remain in integrating diverse data types, minimizing redundancy, and ensuring compatibility across annotation databases. Recognizing these strengths and limitations helps researchers select the most appropriate method for their specific objectives and experimental contexts. The surest way to avoid “pathway fails” is to pair rigorous preprocessing with the interpretation method that best fits the research question: semantic tools for concise GO redundancy reduction, network approaches for interaction context, and embedding strategies for rapid, scalable multi-omic summarization.

We have highlighted areas of improvement for interpretation tools. Ultimately, the principle of “garbage in, garbage out” (GIGO) remains paramount. High-quality, standardized datasets are essential for obtaining reliable results and maximizing the value of any analysis tool [147]. Poor input data or inconsistent standards can significantly limit the effectiveness of these methods and lead to erroneous conclusions, highlighting the need for rigorous data curation and adherence to open data standards. Future developments should focus on enhancing scalability to accommodate increasingly large datasets and ensuring compatibility with so-called non-model model organisms. The influence of gene-set size and database choice on enrichment outcomes must be carefully considered, as this may impact the interpretation of results [148]. Furthermore, consistent standards for functional enrichment analysis, such as proper *p*-value corrections and background gene list selection, are necessary to ensure reliable findings [149,150].

Looking ahead, researchers are encouraged to adopt tools that streamline and automate analysis, thus enhancing reproducibility and scalability. Emerging AI-driven tools are now offering integrative and customizable approaches that potentially reduce misinterpretation by leveraging domain-specific large language models [151,152,153]. These advancements are particularly vital as multi-omics data grow in complexity and require more accurate interpretations. Continued innovation is essential to bridge the gap between computational predictions and experimental validation, driving deeper insights and supporting the advancement of systems biology and precision medicine. As the field evolves, the development of more adaptable, comprehensive, and user-friendly tools will empower researchers to fully leverage omics data, uncover complex biological relationships, and inform therapeutic strategies. By addressing current challenges and promoting methodological rigor, the pathway analysis field will continue to be a robust and indispensable component of modern biological research.

## Figures and Tables

**Table 1:** **Representative top two genes nearest Tukey’s five-number summary of gene ontology annotations (GOALL w/IEA, Bader Lab, October 2024).** The complete list is available in the Supplementary (Table S1).

Gene	# of Pathways
TGFB1 transforming growth factor beta 1	1010
CTNNB1 catenin beta 1	894
ACADL acyl-CoA dehydrogenase long chain	120
ACTBL2 actin beta like 2	120
ABCA6 ATP binding cassette subfamily A member 6	72
ACKR1 atypical chemokine receptor 1 (Duffy blood group)	72
ABCF3 ATP binding cassette subfamily F member 3	44
ADISSP adipose secreted signaling protein	44
C6orf62 chromosome 6 open reading frame 62	2
CTAGE3P CTAGE family member 3, pseudogene	2

**Table 4:** **Summary of pathway interpretation tools by methodological category.** This table groups representative tools by core strategy, with brief notes on their typical strengths and limitations to guide method selection at a glance. For a complete detailed listing of features, see Table 3.

Category	Representative Tools	Typical Strength	Typical Limitation
Semantic similarity-based	REVIGO, clusterProfiler, ReCiPa	Fast redundancy reduction for GO terms	Tied to GO; limited cross-database scope
Network-based	EnrichmentMap, vissE, GScluster	Visualizes pathway crosstalk as network modules	Computationally heavy for large networks
Embedding-based	PAVER, MondrianMap	Data-driven clustering with intuitive plots	Relies on text descriptions; may miss context

## Supplementary Materials

The following supporting information can be downloaded at: https://www.mdpi.com/article/10.3390/biotech14030058/s1.

## Glossary

**Pathway Analysis**	A method for identifying biological pathways enriched with differentially expressed genes or proteins in datasets.
**Dimensionality Reduction**	Techniques used to visualize high-dimensional data in simpler forms for clearer pathway analysis.
**Pathway Redundancy**	The occurrence of overlapping or repeated pathways in analysis, which can complicate interpretation and reduce clarity.
**Embedding-Based Methods**	Computational approaches that represent biological pathways as high-dimensional numerical vectors for analysis.
**Semantic Similarity**	A metric that quantifies the functional similarity between different biological terms or pathways.
**Network-Based Analysis**	A method that visualizes relationships between pathways as interconnected networks, highlighting shared functions or genes.
